# Developing expert international consensus statements for opioid-sparing analgesia using the Delphi method

**DOI:** 10.1186/s12871-023-01995-4

**Published:** 2023-02-27

**Authors:** Daniel Da Der Sng, Giulia Uitenbosch, Hans D. de Boer, Hugo Nogueira Carvalho, Juan P. Cata, Gabor Erdoes, Luc Heytens, Fernande Jane Lois, Paolo Pelosi, Anne-Françoise Rousseau, Patrice Forget, David Nesvadba, Pain AND Opioids after Surgery (PANDOS) European Society of Anaesthesiology, Intensive Care (ESAIC) Research Group, Sadegh Abdolmohammadi, Gebrehiwot Asfaw, Daniel Benhamou, Gilbert Blaise, Philippe Cuvillon, Mohamed El Tahan, Emmanuel Feldano, Paul Fettes, Gabriele Finco, Michael Fitzpatrick, Atul Kapila, Callum Kaye, Vikas Kaura, Helen May, Patrick Meybohm, Ulrike Stamer, Daniel Taylor, Marc Van De Velde, Benoit Van Pee

**Affiliations:** 1 School of Medicine, Medical Sciences and Nutrition, University of Aberdeen, Aberdeen, AB25 2ZD UK; 2 Department of Anesthesiology Pain Medicine and Procedural Sedation and Analgesia, Martini General Hospital Groningen, Groningen, Netherlands; 3 Anesthesiology and Perioperative Medicine, Universitair Ziekenhuis Brussel, Vrije Universiteit Brussel, Jette, Belgium; 4 Department of Anesthesiology and Perioperative Medicine, Division of Anesthesiology, Critical Care, and Pain Medicine, University of Texas MD Anderson Cancer Center, Houston, USA; 5 University Department of Anaesthesiology and Pain Medicine, Inselspital, University Hospital Bern, Bern, Switzerland; 6 Department of Anesthesia, Department of Neurology and Instituut Born-Bunge, University of Antwerp (UA), Antwerpen, Belgium; 7 Centre Hospitalier Universitaire de Liège, Anesthesiology, Liège, Belgium; 8 Department of Surgical Sciences and Integrated Diagnostics (DISC), IRCCS San Martino Policlinico Hospital, Genova GE, Italy; 9 Centre Des Brûlés, Centre Hospitalier Universitaire de Liège, Liège, Belgium; 10 Department of Anaesthesia, Institute of Applied Health Sciences, Epidemiology Group, School of Medicine, Medical Sciences and Nutrition, University of Aberdeen; NHS Grampian, Aberdeen, AB25 2ZD UK; 11 Pain and Opioids After Surgery (PANDOS) European Society of Anaesthesiology and Intensive Care (ESAIC) Research Group, ESAIC, Brussels, Belgium; 12 Department of Anaesthesia, NHS Grampian, Aberdeen, AB25 2ZD UK

**Keywords:** Opioids, Opioid-sparing, Delphi

## Abstract

**Introduction:**

The management of postoperative pain in anaesthesia is evolving with a deeper understanding of associating multiple modalities and analgesic medications. However, the motivations and barriers regarding the adoption of opioid-sparing analgesia are not well known.

**Methods:**

We designed a modified Delphi survey to explore the perspectives and opinions of expert panellists with regard to opioid-sparing multimodal analgesia. 29 anaesthetists underwent an evolving three-round questionnaire to determine the level of agreement on certain aspects of multimodal analgesia, with the last round deciding if each statement was a priority.

**Results:**

The results were aggregated and a consensus, defined as achievement of over 75% on the Likert scale, was reached for five out of eight statements. The panellists agreed there was a strong body of evidence supporting opioid-sparing multimodal analgesia. However, there existed multiple barriers to widespread adoption, foremost the lack of training and education, as well as the reluctance to change existing practices. Practical issues such as cost effectiveness, increased workload, or the lack of supply of anaesthetic agents were not perceived to be as critical in preventing adoption.

**Conclusion:**

Thus, a focus on developing specific guidelines for multimodal analgesia and addressing gaps in education may improve the adoption of opioid-sparing analgesia.

**Supplementary Information:**

The online version contains supplementary material available at 10.1186/s12871-023-01995-4.

## Introduction

Postoperative pain remains, at least partially, an unresolved issue. As approaches to pain management improve, it is clear that opioids alone are not the solution to managing postoperative pain. Over the 21.^st^ century, advancements in the understanding of opioid-based medications, coupled with improved accessibility, have led to more liberal opioid prescriptions. For instance, in the UK, a 34% increase in the prescriptions of opioids was noted between 1998 and 2016 [1]. The over-reliance on opioids has opened the doors to over-prescription, which may lead to worrying outcomes [2]. While they are effective for short-term pain relief, opioids have a propensity to be misused. The costs of over-prescription of opioids have a great economic and human toll, including addiction, dependency, overdose and even death [3–5].

Solutions to mitigating the side effects of opioid over-prescription include increasing the adoption of opioid-sparing approaches to analgesia [6]. A multimodal approach to anaesthesia involves a combination of various opioid-sparing and antinociceptive agents, for example the use of locoregional anaesthesia, alpha-2 agonists and anti-inflammatory drugs [7]. These act on multiple pain pathways and receptors, and may have additive or synergistic effects for pain relief. It is worth noting that multimodal analgesia does not need to be opioid-free, but has the objective of minimising the side effects of opioids and improving pain control. In fact, the Royal College of Anaesthetists (RCoA) made a stand that multimodal analgesia has proven to be opioid-sparing and provides superior pain relief. At present, the RCoA openly encourages the use of opioid-sparing analgesia techniques and opioid-sparing adjuvants [8].

While the multimodal approach is arguably a more complex technique and is currently typically proposed for a niche patient group (e.g., individuals at high risk of moderate-to-severe postoperative pain, sleep-related breathing disorders or pre-existent opioid-related misuse), the assurance of superior pain relief alone invites the question as to why opioid-sparing analgesia is not more widely practiced [9]. Other additional benefits, such as improved patient satisfaction, shortened recovery times and improved pain control in certain surgical procedures, propels the curiosity [10].

To date, there are few published Randomised Controlled Trials (RCTs) related to reasons and opinions of the use of opioid-sparing analgesia. As a result, clinical practice variations in the management of opioid-sparing analgesia have resulted in unclear optimal therapeutic management directions. This may be due to individual preferences and the local practices of anaesthetists. Given the dearth of evidence, the objective of this project is to use a Delphi process to achieve a consensus of the reasons and opinions of the use of multimodal opioid-sparing analgesia.

## Methods

### Methodology model

We employed a modified Delphi survey on opioid-sparing analgesia, which was part of a larger project focusing on reasons behind the selection of specific anaesthetic techniques**.** A modified Delphi survey is broadly defined as a multi-round survey that is anonymised, with a structured information flow and regular feedback [11]. The Delphi technique is often employed to create a robust consensus in healthcare research.

Two primary advantages of the Delphi method are as follows: firstly, it preserves the anonymity of the panellists, allowing for unrestricted expression of opinions, thereby preventing discussion from being dominated by strong personalities. Secondly, regular feedback with aggregated responses from previous rounds creates opportunities for participants to change their minds or admit errors in prior judgements [12]. We hope that establishing a group consensus will lead to stronger conclusions and a more balanced viewpoint. In addition to this, the survey was done remotely, overcoming social distancing restrictions due to COVID-19. This also allowed for a more extensive global reach and a wide range of panellists’ contributions.

**Fig. 1:**
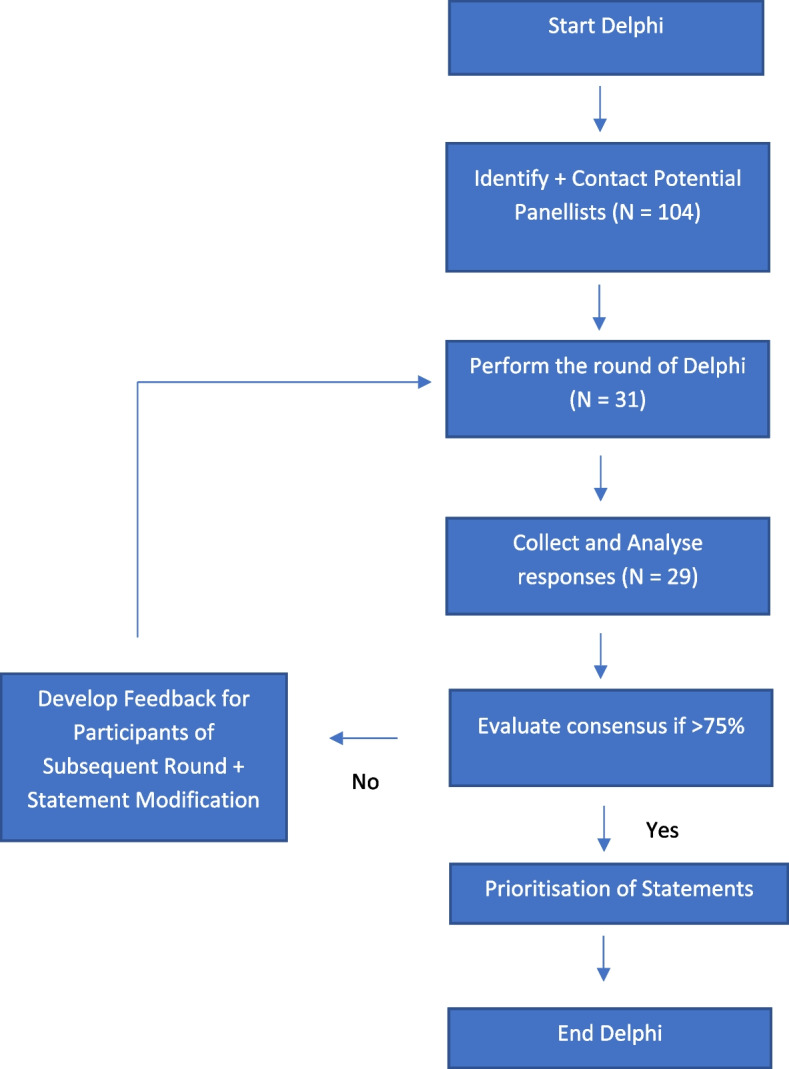
Delphi study flow chart

For the first two rounds, the panellists were presented with eight statements based on our initial research on multimodal analgesia, seeking their perspectives on the strength of evidence as well as potential obstacles to adoption in the post-operative setting [13–15]. They could indicate their level of agreement on a five-point Likert scale. This differs from the classical Delphi approach, which often uses an unstructured first round [16]. The final results of the survey and the expert clinical practice statements were circulated among the experts. The manuscript was then circulated among the experts for editing and approval before it was submitted for publication. Figure 1 details the process of the Delphi study.

### Panellist recruitment

**Fig. 2:**
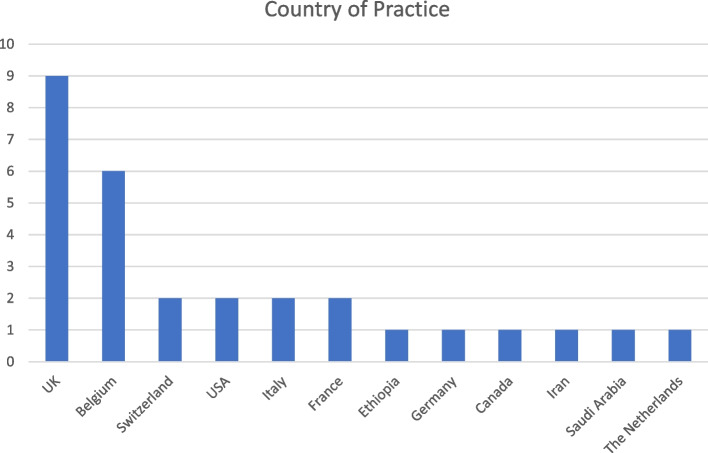
Geographical distribution of countries represented by the experts

We recruited panellists by reaching out to members of the major anaesthetic organisations including the European Association of Cardiothoracic Anaesthesiology and Intensive Care (EACTAIC), European Society of Anaesthesiology and Intensive Care (ESAIC), UK Society for Intravenous Anaesthesia (SIVA), and the European Society for Regional Anaesthesia and Pain Therapy (ESRA). To prevent selection bias, we attempted to gather a diverse group of individuals representing the various subspecialties and anaesthetic organisations. 104 anaesthetic organisations and expert individuals were contacted for representatives who would be keen on participating in the survey, and the geographical distribution of the 29 panellists are shown in Fig. 2 below. As opinions on opioid-sparing techniques may vary between geographical locations and professional settings, the panellists were informed beforehand that they did not represent their respective organisations, but rather individual perspectives based on their personal experience.

### Data collection structure

A total of 31 panellists responded positively to complete the survey. Study data was collected and managed using REDCap (Research Electronic Data Capture) electronic data capture tools hosted at the University of Aberdeen. REDCap is a secure, web-based application designed to support data capture for research studies, providing: 1) an intuitive interface for validated data entry; 2) audit trails for tracking data manipulation and export procedures; 3) automated export procedures for seamless data downloads to common statistical packages; and 4) procedures for importing data from external sources [17]. The application ensured the anonymity of data collection, provided a structured framework for providing feedback to the panellists, and facilitated the sending of automated emails and reminders for each Delphi round.

### Data collection process

In Round 1, the panellists were requested to select their level of agreement based on a 5-point Likert scale, ranging from ‘Not at all’ to ‘Very much’. Using free-text format, panellists were also invited to propose new statements describing what they felt would be the largest obstacles to the adoption of opioid-sparing analgesia. These new statements were taken into consideration and incorporated into the statement pool in the second round. Additionally, specific panellist demographics were collected (e.g. country of practice, years of practice since qualification, areas of interest) which are partially included in Table 1.

**Table 1:** Panellist profile

Category	No of Panellists
**Hospital Grade**	
Tertiary	21 (72.4%)
Secondary	8 (27.6%)
**Years of Anaesthetic practice**	
0–10	6 (20.6%)
11–20	12 (41.4%)
> 20	11 (37.9%)

In the subsequent second and third rounds, panellists were presented their initial choices in the context of the aggregated and anonymised responses of all panellists, and thereafter given the choice to change their selection or retain the same position.

In the third round, the statements were modified to determine if the respective elements were a priority in the use of opioid-sparing analgesia via a Yes/No question format. Notably, one statement which experienced a greater than 10% change from the previous round was retained in the survey to ensure the stability of the consensus.

### Data analysis

A consensus was defined as 75% of panellists agreeing somewhat/very much or disagreeing not much/not at all. This is a commonly accepted threshold within Delphi studies [18]. Stability was taken as attained if the variation between each Delphi round was 10% or less.

## Results

### Round 1

Twenty-nine out of the 31 invited panellists participated in the Delphi survey. There were two individuals who initially accepted the invitation to participate but eventually did not respond. Panellists indicated their country of practice, number of years of practice, as well as the professional setting where they practice. Eight items were presented. All 29 panellists completed the survey and 24 provided additional comments.

### Round 2

All 29 panellists that completed Round 1 participated in Round 2. Based on the feedback in Round 1, three out of the eight statements were modified according to their inputs. The final statements are detailed in Table 2.

**Table 2:** Statements and results

		**Final Agreement**	**% change**
1	There is a strong body of evidence supporting the use of opioid-sparing techniques	79.3%	+ 6.9%
2	Whether opioid-sparing techniques may be cost effective is an important aspect for me	51.7%	+ 6.9%
3	Whether opioid-sparing techniques and/or multimodal analgesia is the norm in my context and/or recommended in the locally used guidelines is important in my practice	82.6%	0%
4	The lack of training/education for some techniques possibly useful in multimodal analgesia is a key reason anaesthesiologists may not use it	92.6%	+ 6.4%
5	I feel confident in administering any opioid sparing technique I need	75.8%	0%
6	Leadership and/or more specific guidelines for the application of multimodal analgesia will help my practice	79.3%	+ 10.3%
7	The use of multimodal analgesia, or opioid-sparing techniques, is impractical (time consuming/workload) in my practice (whatever the reason)	10.3%	0%
8	The lack of supply of certain analgesic agents restricts my practice of multimodal analgesia	34.5%	0%

### Round 3

Twenty-eight out of 29 panellists from Round 2 participated in Round 3. We used the predetermined 75% agreement threshold to determine which of the statements to prioritise. Following this, there were five questions to prioritise, and the level of agreement ranged from 85.7% to 96.4%. The results are detailed in Table 3.

**Table 3:** Prioritisation of statements (sorted by highest percentage)

Statements	Prioritisation in Agreement
Do you think training/education is a priority for the use of opioid-sparing/multimodal analgesia?	96.4%
Do you think confidence in administering opioid sparing techniques is a priority that determines the use of opioid-sparing analgesia?	96.4%
Do you think the strength of evidence is a priority in determining the use of opioid-sparing analgesia?	89.3%
Do you think more leadership and more specific guidelines for the application of multimodal analgesia is a priority for the adoption of multimodal analgesia?	89.3%
Do you think the locally adopted practices and recommendations are a priority when determining the use of opioid-sparing analgesia/multimodal analgesia?	85.7%

## Discussion

The Delphi survey reached a strong agreement on five different statements, which were all considered by the panellists as priorities with regard to the adoption of opioid-sparing analgesia. With each survey round, we gained insight into the perspectives of anaesthetists who seemed to be generally in favour of opioid-sparing/multimodal analgesia. There was a strong consensus that there is a robust body of evidence behind the multimodal analgesic approach, with 79.3% in agreement. 89.3% of the panellists believed that the strength of evidence is a priority determining the use of opioid-sparing analgesia.

However, there appear to be significant barriers to widespread adoption. At 92.4%, the lack of training and education reached the strongest consensus that it was likely a key factor preventing anaesthetists from using multimodal analgesia. Similarly, this gap in education was identified as the highest priority that determined the use of opioid-sparing analgesia, at 96.4%. This correlated to the results of the first round, where 41.4% panellists commented that inadequate knowledge and training for these techniques was the main obstacle in the adoption of multimodal analgesia. These findings suggest that since it is believed that there is a strong evidence base for opioid-sparing analgesia, more should be done to introduce these techniques into specialty training programs to build up experience. Gaps in the curriculum could be identified and addressed, with a greater emphasis on multimodal analgesia.

Despite identifying the lack of education as an obstacle, the panellists themselves felt confident in administering opioid sparing analgesia, with 75.8% of panellists believing they could administer any technique they may require. This may be partially explained by the panellist selection process, which included many experienced leaders in their field. 79.4% of panellists had over 10 years of practice after full qualification. This finding highlights that while training does exist for multimodal anaesthetic techniques, it is presently not accessible to everyone. Facilitating access and education may thus lead to a better application of current guidelines and improve patient outcomes for pain relief.

One recommendation from the panellists was that more leadership and specific guidelines for multimodal analgesia could increase the adoption of these techniques, with 89.3% of the participants believing it should be a priority. There was a 79.3% consensus that more specific guidelines and leadership would improve their own practice. More structured administration procedures and clear communication may be a potential means to encourage more healthcare professionals to adopt less mainstream techniques and build confidence in expanding their range of skills.

Interestingly, despite initial research postulating that cost may be an obstacle for multimodal analgesia as compared to opiate-based drugs, our survey did not surface a consensus to suggest so [19]. Only 51.7% of panellists agreed with the statement that the cost-effectiveness of opioid-sparing techniques was of importance to them. Likewise, the logistical complications of opioid-sparing techniques, such as technique and the lack of supply of analgesic agents, were not considered to be a major factor restricting practice (34.5% of panellists in agreement). This finding may be attributed to differences arising from the structure of healthcare systems the panellists practice in, given that a majority are from the UK and Belgium. To reduce potential inequities, this area merits further exploration for a deeper understanding of the intricacies.

Another noteworthy finding is that the panellists deemed multimodal opioid-sparing analgesia as feasible to put into practice. There was a strong consensus of 79.3% disagreeing that multimodal analgesia was impractical (e.g. more time consuming, creating a greater workload for anaesthetists). These practical aspects thus seem satisfactory at present, and may not require large restructuring and investment.

Apart from our pre-determined statements, the survey uncovered other factors that may warrant further investigation. Panellists identified several barriers to the widespread uptake of multimodal analgesia in the free-text portion of Round 1 – for example, 24.1% of panellists raised concerns surrounding the reluctance to change their practice and resistance to disrupting the status quo. As these barriers are more deep-seated in nature, a local or national project vis-à-vis an international initiative may tackle them more incisively. Raising awareness of the benefits of and evidence backing multimodal opioid-sparing analgesia whilst addressing localised concerns may kickstart the gears of change.

### Limitations

The study was not without its limitations. Firstly, it was challenging to fully integrate all inputs from the free-text portion of the survey. This is a common shortcoming associated with the brevity and potential lack of clarity of online platforms. While discussions may be less rich than a focus group format, the online Delphi method was more practical and international-reaching. It also facilitated more robust discussion as survey responses were kept anonymous.

Secondly, certain survey questions were flagged by panellists as being open to interpretation. This may be due to the international nature of the project, with a significant proportion of panellists being non-native English speakers.

Lastly, a large proportion of panellists were from the UK and Belgium despite our best efforts to have a wide selection of international participants for adequate representation of geographic differences and subspecialties. The panel further composed mostly of anaesthetists at the mid- to late- career stage, with the overall panellist number tending towards the smaller bound. While these factors may limit the generalisability of the findings, they simultaneously draw attention to other potential differences in perspectives on barriers to adopting multimodal analgesia plausibly attributed to recent educational shifts in anaesthesia techniques. This suggests an interesting comparison for future exploration.

## Conclusion

This project explored the perspectives of anaesthetists regarding opioid-sparing analgesia. Practical considerations appeared to be less of an obstacle, as cost effectiveness, practicalities of time management or an increased workload were not regarded as significant problems. However more deep-seated and time-resistant barriers still remain, such as the reluctance to adopt new practices and reform existing practices. The lack of leadership, education, and training were also identified as obstacles. Evidently, developing specific guidelines for multimodal analgesia and addressing gaps in education are critical first steps to encourage the general adoption of opioid-sparing analgesia.

## Supplementary Information

**Additional file 1.**

## Data Availability

All data generated or analysed during this study are included in this published article [and its supplementary information files].
