# Supplementary material for: Developing expert international consensus statements for opioid-sparing analgesia using the Delphi method
Source: BMC Anesthesiol. 2023 Feb 27;23:62. doi: 10.1186/s12871-023-01995-4 (PMC9969386; doi:10.1186/s12871-023-01995-4)
Supplement: Supplementary file 1 — Additional file 1. [file 12871_2023_1995_MOESM1_ESM.docx]

**Appendix 1. Statements in Round 1 and 2 of the Delphi Survey**

This table describes the 8 statements including modifications in italics and the percentage of agreement reached after each round. Consensus was considered reached if above 75%, and they are presented in bold.

| Statement 1 | Round 1: There is a strong body of evidence supporting the use of opioid sparing techniques. | 72.4% |
| --- | --- | --- |
|  | Round 2: Statement unmodified. | **79.3%** |
| Statement 2 | Round 1: Whether opioid sparing techniques may be cost effective is an important aspect for me. | 44.8% |
|  | Round 2: Statement unmodified. | 51.7% |
| Statement 3 | Round 1: Whether opioid sparing techniques and/or multimodal analgesia is the norm in my context and/or recommended in the locally used guidelines is important in my practice. | **82.6%** |
|  | Round 2: Statement unmodified. | **82.6%** |
| Statement 4 | Round 1: The lack of training on some techniques possibly useful in multimodal analgesia is a key reason why I, or colleagues I know, do not use it. | 68.9% |
|  | Round 2: The lack of training/*education* for some techniques possibly useful in multimodal analgesia is a key reason anaesthesiologists *may not* use it. | **86.2%** |
|  | Round 3: The lack of training/education for some techniques possibly useful in multimodal analgesia is a key reason anaesthesiologists may not use it. | **92.6%** |
| Statement 5 | Round 1: I feel confident in administering any opioid sparing technique I need. | **75.8%** |
|  | Round 2: Statement unmodified. | **75.8%** |
| Statement 6 | Round 1: More specific guidelines for the application of multimodal analgesia will help my practice. | 69.0% |
|  | Round 2: *Leadership and/or* more specific guidelines for the application of multimodal analgesia will help my practice. | **79.3%** |
| Statement 7 | Round 1: The use of multimodal analgesia, or opioid sparing techniques, is impractical in my practice (whatever the reason). | **75.9%** |
|  | Round 2: The use of multimodal analgesia, or opioid sparing techniques, is impractical *(time consuming/workload)* in my practice (whatever the reason). | **79.3%** |
| Statement 8 | Round 1: The lack of supply of certain analgesic agents restricts my practice of multimodal analgesia. | 37.9% |
|  | Round 2: Statement unmodified | 34.5% |

**Appendix 2: Panellist Profile**

| **Areas of Interest (n = 51)** |  |
| --- | --- |
| General Anaesthesia | 5 (9.8%) |
| Regional Anaesthesia | 5 (9.8%) |
| Critical Care | 5 (9.8%) |
| Neuroanaesthesia | 4 (7.8%) |
| Cardiac Anaesthesia | 4 (7.8%) |
| Pain Medicine | 4 (7.8%) |
| Obstetric Anaesthesia | 2 (3.9%) |
| Non-Operating Room Anaesthesia | 2 (3.9%) |
| Education | 2 (3.9%) |
| Paediatric Anaesthesia | 2 (3.9%) |
| Malignant Hyperthermia | 2 (3.9%) |
| Orthopaedic Anaesthesia | 2 (3.9%) |
| Airway | 1 (1.9%) |
| Burn Care | 1 (1.9% |
| Vascular Anaesthesia | 1 (1.9%) |
| Opioid Free Anaesthesia | 1 (1.9%) |
| Quality and Safety | 1 (1.9%) |
| Transfusion | 1 (1.9%) |
| Myopathies | 1 (1.9% |
| Monitoring | 1 (1.9%) |
| Cardiothoracic Anaesthesia | 1 (1.9%) |
| Cancer | 1 (1.9%) |
| Unspecified | 2 (3.9%) |
